# A Bidirectional Label Propagation Based Computational Model for Potential Microbe-Disease Association Prediction

**DOI:** 10.3389/fmicb.2019.00684

**Published:** 2019-04-09

**Authors:** Lei Wang, Yuqi Wang, Hao Li, Xiang Feng, Dawei Yuan, Jialiang Yang

**Affiliations:** ^1^Key Laboratory of Hunan Province for Internet of Things and Information Security, Xiangtan University, Xiangtan, China; ^2^College of Computer Engineering and Applied Mathematics, Changsha University, Changsha, China; ^3^Geneis Beijing Co., Ltd., Beijing, China

**Keywords:** microbe-disease association, bidirectional label propagation, leave-one-out cross validation, 5-fold cross validation, COPD

## Abstract

A growing number of clinical observations have indicated that microbes are involved in a variety of important human diseases. It is obvious that in-depth investigation of correlations between microbes and diseases will benefit the prevention, early diagnosis, and prognosis of diseases greatly. Hence, in this paper, based on known microbe-disease associations, a prediction model called NBLPIHMDA was proposed to infer potential microbe-disease associations. Specifically, two kinds of networks including the disease similarity network and the microbe similarity network were first constructed based on the Gaussian interaction profile kernel similarity. The bidirectional label propagation was then applied on these two kinds of networks to predict potential microbe-disease associations. We applied NBLPIHMDA on Human Microbe-Disease Association database (HMDAD), and compared it with 3 other recent published methods including LRLSHMDA, BiRWMP, and KATZHMDA based on the leave-one-out cross validation and 5-fold cross validation, respectively. As a result, the area under the receiver operating characteristic curves (AUCs) achieved by NBLPIHMDA were 0.8777 and 0.8958 ± 0.0027, respectively, outperforming the compared methods. In addition, in case studies of asthma, colorectal carcinoma, and Chronic obstructive pulmonary disease, simulation results illustrated that there are 10, 10, and 8 out of the top 10 predicted microbes having been confirmed by published documentary evidences, which further demonstrated that NBLPIHMDA is promising in predicting novel associations between diseases and microbes as well.

## 1. Introduction

With the development of sequencing technologies, studies on microbes in soils, oceans, human bodies and other places have received increasing attention from the scientific community (Methé et al., 2012). Human microbiota, i.e., the collection of microbes existing in human tissues and biological fluids, includes various species such as archaea, eukaryotes, bacteria, and viruses. It is known that all parts of human body contain microbes, the number of which is more than 10 times the number of cells in a human body (Turnbaugh et al., 2007; Sender et al., 2016). Fortunately, the vast majority of microbes are harmless to human body, some of which are even indispensable for our metabolism, growth, and development. For example, there are parasitic microbes involving in the physiological mechanisms of the human body and playing a vital role in the process of energy acquisition and storage, salvage of energy, and nutrient, resistance to pathogens and foreign microorganisms, immune responses, and other metabolic processes (Guarner and Malagelada, 2003). As a result, human health will be greatly affected by the human microbiota, and the disorder and imbalance of them will sometimes lead to diseases.

The microbiota has been living and evolving in human body since the emerging of human beings, and they have gradually formed a close symbiotic relationship. The dynamic balance of microbiota and human diseases has become a hot topic recently. For example, a few studies indicate that the host's diet affects the activity and structure of microbiota in the human gut. Long-term high-fat diets can cause changes in the intestinal microbiota, resulting in an increase in intestinal deoxycholic acid concentration (DCA), which may promote the development of liver cancer (David et al., 2013). In addition, researchers also found that smoking creates a microenvironment in which a dangerous microbiota is produced, and reducing the niche saturation of the microbiota will increase the risk of diseases (Mason et al., 2014).

The microbiota can also be affected by genes (Khachatryan et al., 2008; Turnbaugh et al., 2008; Goodrich et al., 2014), seasons (Davenport et al., 2014), hygiene and antibiotics (Donia et al., 2014), and so on. With the advent of high-throughput methods and advanced analytical techniques, the collective genome of microbes (human microbiome) inhabiting the human body has become a key player in human health and diseases. Based on the high-resolution molecular analysis, a few researchers found that the disorder of microbiota in the gastrointestinal tract is closely associated with several idiopathic diseases, such as diabetes, obesity, inflammatory bowel disease, cancer, kidney stones, and neurodegenerative diseases (Shah et al., 2016). For example, according to the study of inflammatory bowel disease, the intestinal flora is found to be able to regulate the inflammatory response by regulating regulatory T cells (Singh et al., 2001). In addition, as pointed out by Christopher et al., microbiota that produces lactic acid and butyl hydrochloric acid such as Akkermansia and Prevotella can induce the synthesis of intestinal mucin, which may contribute to intestinal health (Brown et al., 2011). Furthermore, microbiota has been reported to play an important role in autoimmune diseases such as type 1 diabetes. For example, a population of dysfunctional microbes were found in subjects immunized with type 1 diabetes (Murri et al., 2013).

As disease-associated flora can provide important insights into the understanding of the formulation and development of diseases, a few related large-scale projects including the Human Microbiome Project (HMP) (Turnbaugh et al., 2007) and the Earth Microbiome Project (EMP) (Gilbert et al., 2010) have been launched to entangle the relationship between microbial flora and diseases. In addition, many useful databases have also been developed to curate disease-related microbial information. For example, Ma et al. sorted out the confirmed microbe-disease association from published literatures through large-scale text mining, and established the Human Microbe-Disease Association Database (HMDAD) (Ma et al., 2016). These datasets can be served as valuable sources for predicting novel microbe-disease associations.

Traditional microbial identification are performed mainly by independent culture methods and quantitative methods, which are costly and labor intensive. This presents the need for more effective computational methods to scale down the potential microbe-disease associations for further experimental validation. In fact, similar computational models have been successfully implemented in many other related fields such as drug-target interaction prediction (Chen et al., 2012), gene-disease association prediction (Zou, 2016; Meng et al., 2017; Zeng et al., 2017; Zhu et al., 2018), lncRNA-disease association prediction (Chen et al., 2016c; Yu et al., 2018), miRNA-disease relationship prediction (Zeng et al., 2015, 2018; Tang et al., 2017; You et al., 2017), protein structure prediction, and so on. For the first time, Chen et al. proposed the KATZHMDA model for measuring the human microbe-disease association based on KATZ method (Chen et al., 2016a). KATZHMDA first constructed a heterogeneous network composed of the microbe-disease association network, the disease similarity network and the microbe similarity network, and introduced the concept of variable step number to predict microbe-disease associations, which achieves reliable prediction performance. Subsequently, Shen et al. adopted a random walk with restart algorithm to score each candidate microbe-disease pair on a heterogeneous network composed of the Spearman correlation-based microbe network, the symptom-based disease network and the microbe-disease association network (Shen et al., 2016). Huang et al. proposed a path-based computational model called PBHMDA, which adopts a special depth-first search algorithm to traverse all possible paths between microbes and diseases in the heterogeneous network (Huang et al., 2017b). Wang et al. put forward a semi-supervised model of Laplacian regularized least squares (LRLSHMDA), which utilizes Laplace's regular least squares classification combined with topological information of the known microbe-disease association network to train an optimal classifier (Wang et al., 2017). Based on the Gaussian kernel similarity and symptom-based similarity, Huang et al. presented NGRHMDA combining the neighbor-based collaborative filtering model and the bipartite graph-based prediction model (Huang et al., 2017a). Shen et al. developed Bi-Random Walk based on Multiple Path with different length of path (BiRWMP) to predict microbe-disease associations (Shen et al., 2018).

In this paper, we have proposed a novel computational model called NBLPIHMDA based on the bidirectional label propagation to predict potential microbe-disease associations. NBLPIHMDA first calculates the disease similarity matrix and the microbe similarity matrix by introducing the Gaussian interaction profile kernel similarity, based on which a disease similarity network and a microbe similarity network are constructed simultaneously. After that, the edge weights of nodes in these two networks are calculated by using the Gaussian interaction profile kernel similarity. Finally, the bidirectional label propagation is performed on these two weighted networks to obtain the correlation score matrix between diseases and microbes. The final prediction results are obtained by integrating these two correlation score matrices. The leave-one-out cross validation (LOOCV) and 5-fold cross validation (5-fold CV) are adopted to evaluate the predictive performance. As a result, NBLPIHMDA can achieve the area under the receiver operating characteristic (ROC) curves (AUCs) of 0.8777 and 0.8958 ± 0.0027, respectively. In addition, case studies on asthma, colorectal carcinoma and Chronic obstructive pulmonary disease (COPD) further demonstrate that NBLPIHMDA can be considered as an effective tool to discover reliable pathogenic microbes in the future.

## 2. Materials and Methods

We downloaded known microbe-disease associations from the Human Microbe-Disease Association database (HMDAD on http://www.cuilab.cn/hmdad), which contains 483 microbe-disease associations including 39 diseases and 292 microbes collected from 61 publications (Ma et al., 2016). After removing redundant associations, we finally obtained 450 distinct microbe-disease associations (Chen et al., 2016a). Based on these associations, we constructed a 39 × 292 dimensional adjacency matrix *A* as our data source, where *A*(*i, j*) = 1 if and only if there is a known association between the disease i and the microbe j, and *A*(*i, j*) = 0 otherwise. For convenience, we denote the number of collected diseases by *N*_*D*_ and the number of collected microbes by *N*_*M*_.

### 2.1. Diseases Similarity Based on Gaussian Interaction Profile Kernel Similarity

Based on the assumption that diseases related to similar microbes tend to have more functional similarity and share similar interaction and non-interaction patterns with microbes (Chen et al., 2016a), we calculated the Gaussian interaction profile kernel similarity between each pair of diseases by using the Gaussian kernel for the interaction profiles of them. Specifically, for any two given diseases *d*_*i*_ and *d*_*j*_, their Gaussian interaction profile kernel similarity can be calculated as follows:

(1) $$SD(d_{i},d_{j})=exp(-\gamma_{d}{\left\|IP(d_{i})-IP(d_{j})\right\|}^{2})$$

Where the interaction profile *IP*(*d*_*t*_) indicates whether there is an association between disease *d*_*t*_ and each microbe, and it is defined as a binary vector, i.e., the *t*^*th*^ row of the adjacency matrix *A*. ||*IP*_*d*_*t*__|| represents the norm of the binary vector *IP*(*d*_*t*_). The parameter γ_*d*_ is used to control the kernel bandwidth, which needs to be calculated by normalizing a new bandwidth parameter ${\gamma^{\prime }}_{d}$ according to the average number of associations between each disease and microbes (Chen et al., 2016a). The calculation formula is as follows:

(2) $$\gamma_{d}={\gamma^{\prime }}_{d}/(\frac{1}{N_{D}}\sum_{k=1}^{N_{D}}\;{\left\|\;IP(d_{k})\right\|}^{2})$$

Although it may be possible to set the new bandwidth parameter ${\gamma^{\prime }}_{d}$ to a better value by cross validation experiments, in this paper we will set ${\gamma^{\prime }}_{d}$ to 1 for the sake of simplicity. Hence, based on above formulas, we can finally obtain a matrix *SD*, where *SD*(*i, j*) represents the score of the Gaussian interaction profile kernel similarity between diseases *d*_*i*_ and *d*_*j*_.

### 2.2. Microbes Similarity Based on Gaussian Interaction Profile Kernel Similarity

Similar to the way presented in above section 3.1, based on the assumption that microbes related to similar diseases tend to show more functional similarity and share similar interaction and non-interaction patterns with diseases (Chen et al., 2016a), the Gaussian interaction profile kernel similarity between each pair of microbes can be computed according to the following formula (3) as well:

(3) $$SM(m_{i},m_{j})=exp(-\gamma_{m}{\left\|IP(m_{i})-IP(m_{j})\right\|}^{2})$$

Where the interaction profile *IP*(*m*_*t*_) indicates whether there is an association between microbe *m*_*t*_ and each disease, and it is defined as a binary vector, i.e., the *tth* column of the adjacency matrix *A*. The parameter γ_*m*_ utilized to control the kernel bandwidth can be calculated by normalizing a new bandwidth parameter ${\gamma^{\prime }}_{m}$ as follows:

(4) $$\gamma_{m}={\gamma^{\prime }}_{m}/(\frac{1}{N_{M}}\sum_{k=1}^{N_{M}}{\left\|IP(m_{k})\right\|}^{2})$$

And in this paper, the new bandwidth parameter ${\gamma^{\prime }}_{m}$ will be set to 1 for the sake of simplicity as well. Hence, based on above formulas, we can finally obtain another matrix *SM*, where *SM*(*i, j*) represents the score of the Gaussian interaction profile kernel similarity between microbes *m*_*i*_ and *m*_*j*_.

### 2.3. Constructing Weighted Networks for Diseases and Microbes

According to these two kinds of Gaussian interaction profile kernel similarity score matrices *SM* and *SD* calculated above, it is obvious that we can construct a microbe similarity network based on *SM* and a disease similarity network based on SD, respectively. Moreover, considering that the similarity values between any two diseases or microbes calculated by the Gaussian kernel for the interaction profiles will not be zero, therefore it is obvious that both the newly constructed microbe similarity network and disease similarity network will be fully connected networks.

Additionally, while implementing the label propagation method (Zhu and Ghahramani, 2002) on the newly constructed disease similarity network, for any given disease node *d*_*i*_, we will assign a initial label *A*(*i*, :) to *d*_*i*_ first, where *A*(*i*, :) represent the *i*^*th*^ row of the adjacency matrix *A* constructed in above section 2. And then, these label information can be propagated between neighboring nodes in the disease similarity network, thereafter each node can update its label information according to the label information received from its neighboring nodes. However, while updating its label information, it is reasonable to the node that its label information should be updated according to these neighboring nodes with high similarity to it rather than all of its neighboring nodes. And moreover, these neighboring nodes with higher similarity to it should be assigned larger weights in the process of updating as well. Hence, based on above analysis, for any given disease node *d*_*i*_, let *Q*_*d*_ represent the set of disease nodes other than *d*_*i*_ itself and these *K* disease nodes with the top *K* lowest similarity to *d*_*i*_, then we can a novel matrix SD^*^ based on above obtained matrix *SD* as follows:

(5) $$SD^{*}(d_{i},d_{j})=\{\begin{array}{c} \frac{SD(d_{i},d_{j})}{\sum_{d_{k}\in Q_{d}}\;SD{\left(d_{i},d_{k}\right)}^{\prime }},if\;d_{j}\in Q_{d}\\ 0,\;otherwise \end{array}$$

Furthermore, in a similar way, while implementing the label propagation method on the newly constructed microbe similarity network, for any given microbe *m*_*i*_, we will assign a initial label *A*(:, *i*) to *m*_*i*_ first, where *A*(:, *i*) represent the *i*^*th*^ column of the adjacency matrix *A* constructed in above section 2. And thereafter, let *Q*_*m*_ represent the set of microbe nodes other than *m*_*i*_ itself and these *K* microbe nodes with the top *K* lowest similarity to *m*_*i*_. then we can a novel matrix SM^*^ based on above obtained matrix *SM* as follows:

(6) $$SM^{*}(m_{i},m_{j})=\{\begin{array}{c} \frac{SM(m_{i},m_{j})}{\sum_{m_{k}\in Q_{m}}\;SM{\left(m_{i},m_{k}\right)}^{\prime }},if\;m_{j}\in Q_{m}\\ 0,\;otherwise \end{array}$$

Thus, according to above formula (5) and formula (6), we can further construct a updated disease similarity network and a updated microbe similarity network based on these two kinds of newly obtained matrices such as SD^*^ and SM^*^. Thereafter, in this way, we have built two novel weighted networks that are adapted to label propagation.

### 2.4. NBLPIHMDA

**Figure 1:**
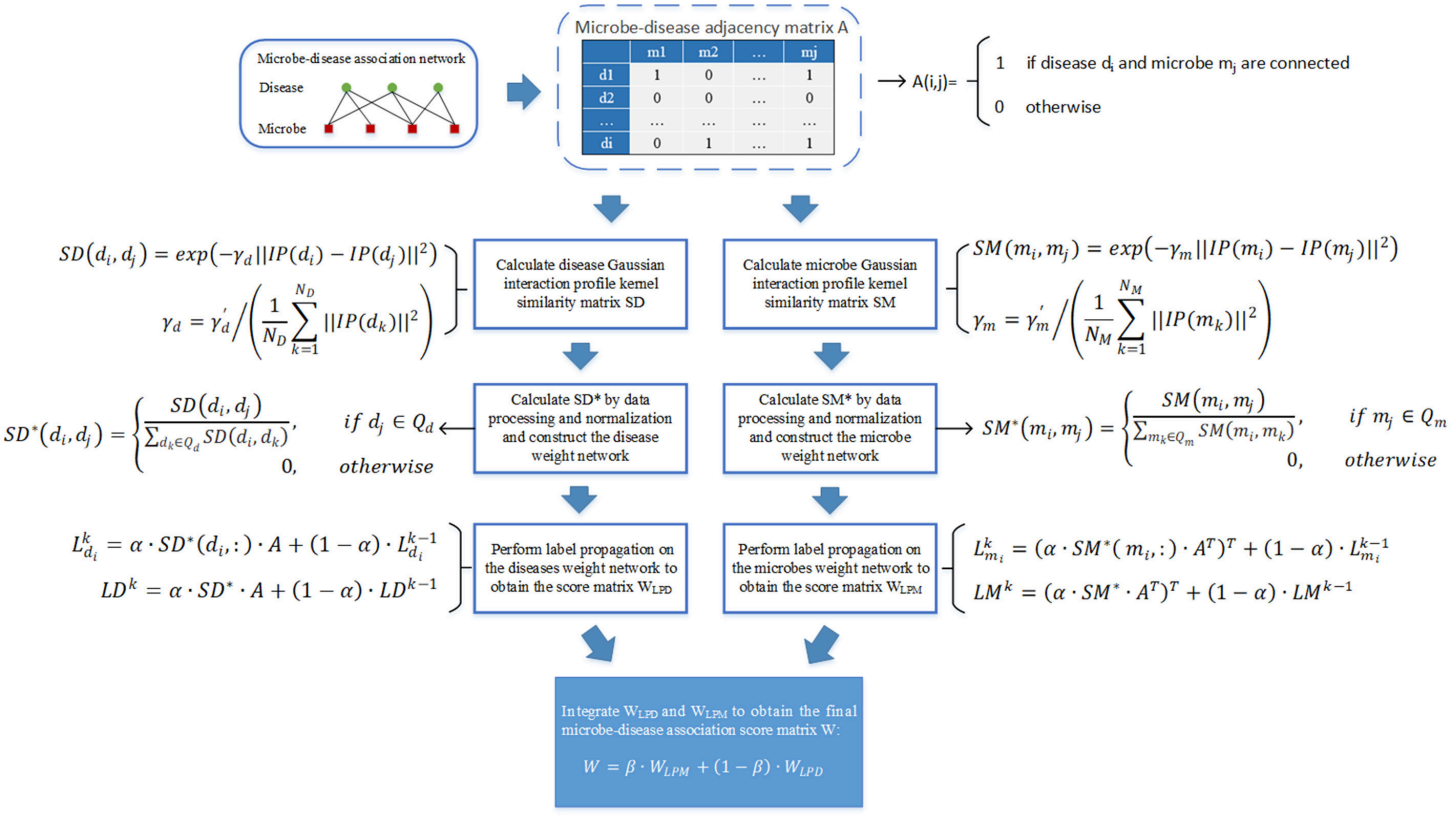
The flowchart of NBLPIHMDA.

As illustrated in the following Figure 1, the implementation process of our prediction model NBLPIHMDA can be divided into the following major steps.

Step 1: Firstly, we will implement the label propagation on the updated disease similarity network, for each given disease node *d*_*i*_, supposing that its label information is updated by absorbing the labels from its neighboring nodes with probability α and retaining its previous label with probability 1−α, and in addition, let $L_{d_{i}}^{0}=A\left(i,:\right)$ denote the initial label of the node *d*_*i*_, $L_{d_{i}}^{k}$ represent the label of *d*_*i*_ after *k* rounds of updating, then $L_{d_{i}}^{k}$ can be calculated as follows:

(7) $$L_{d_{i}}^{k}=\alpha \;\cdot \;SD^{*}(d_{i},:)\;\cdot \;A+(1-\alpha )\;\cdot \;L_{d_{i}}^{k-1}$$

Moreover, as for all disease nodes, supposing that their label vectors be $L_{d_{1}}^{k},L_{d_{2}}^{k},\cdots \;,L_{d_{N_{D}}}^{k}$ after *k* rounds of updating in the updated disease similarity network, and for convenience, let $LD^{k}=\left(L_{d_{1}}^{k};L_{d_{2}}^{k};\cdots \;;L_{d_{N_{D}}}^{k}\right)$, then, we can rewrite above formula (7) into the following form of matrix:

(8) $$LD^{k}=\alpha \;\cdot \;SD^{*}\;\cdot \;A+(1-\alpha )\;\cdot \;LD^{k-1}$$

According to above (8), the labels of each disease node in the updated disease similarity network can be updated iteratively. And during the process of iterative updating, we will consider the iteration to be convergent and stop the process of iterative updating while the change between the updated label matrix *LD*^*k*^ and the former label matrix *LD*^*k*−1^ measured by the absolute loss function is less than a predetermined threshold *P*. Thus, supposing that the process of iterative updating stopped after *n*1 rounds of iterations, we can obtain a microbe-disease association score matrix *W*_*LPD*_ as follows:

(9) $$W_{LPD}=LD^{0}+LD^{1}+...+LD^{n_{1}}$$

Step 2: Next, we will implement the label propagation on the updated microbe similarity network, for each given microbe node *m*_*i*_, supposing that its label information is updated by absorbing the labels from its neighboring nodes with probability α and retaining its previous label with probability 1−α, and in addition, let $L_{m_{i}}^{0}=A\left(:,i\right)$ denote the initial label of the node *m*_*i*_, $L_{m_{i}}^{k}$ represent the label of *m*_*i*_ after *k* rounds of updating, then $L_{m_{i}}^{k}$ can be calculated as follows:

(10) $$L_{m_{i}}^{k}={\left(\alpha \;\cdot \;SM^{*}(m_{i},\;:)\;\cdot \;A^{T}\right)}^{T}+(1-\alpha )\;\cdot \;L_{m_{i}}^{k-1}$$

Moreover, as for all microbe nodes, supposing that their label vectors be $L_{m_{1}}^{k},L_{m_{2}}^{k},\cdots \;,L_{m_{N_{M}}}^{k}$ after *k* rounds of updating in the updated disease similarity network, and for convenience, let $LM^{k}=(L_{m_{1}}^{k},L_{m_{2}}^{k},\cdots ,L_{m_{N_{M}}}^{k})$, then, we can rewrite above formula (10) into the following form of matrix:

(11) $$LM^{k}={\left(\alpha \;\cdot \;SM^{*}\cdot A^{T}\right)}^{T}+(1-\alpha )\;\cdot \;LM^{k-1}$$

Thus, supposing that the process of iterative updating stopped after *n*2 rounds of iterations, then in a similar way, we can obtain another microbe-disease association score matrix *W*_*LPM*_ as follows:

(12) $$W_{LPM}=LM^{0}+LM^{1}+...+LM^{n_{2}}$$

Finally, through combining these two kinds of score matrices *W*_*LPD*_ and *W*_*LPM*_ obtained by bidirectional label propagation, we can ultimately obtain a final microbe-disease association score matrix *W* as follows:

(13) $$W=\beta \;\cdot \;W_{LPM}+(1-\beta )\;\cdot \;W_{LPD}$$

Here, β is a parameter with value between 0 and 1 for controlling the weights of *W*_*LPD*_ and *W*_*LPM*_.

## 3. Results

### 3.1. Performance Evaluation

We adopted LOOCV and 5-fold CV to evaluate the performance of NBLPIHMDA. In the framework of LOOCV, each known microbe-disease association was taken as the test sample in turn, while the remaining known associations were taken as the training set. In addition, all microbe-disease pairs without known associations would be considered negative samples. Thereafter, based on the predicted scores, each test sample would be ranked with all microbe-disease pairs that were not confirmed to be associated. Samples with rankings above the given threshold were predicted to be positive, whereas samples with rankings below the given threshold were predicted to be negative. In addition, test samples with rankings above the given threshold were considered to be successful samples. Next, in the framework of 5-fold CV, known microbe-disease associations were randomly divided into five groups, and each group was selected as a test sample in turn, while the remaining four groups were used as training samples. In order to reduce the deviation caused by random grouping, this process would be performed 100 times. Under the setting of different thresholds, the ROC curve could be further plotted by calculating the corresponding true and false positive rates. In our experiments, sensitivity referred to the percentage of positive samples with rankings above the given threshold, and the specificity was the percentage of negative samples with rankings below the given threshold. Subsequently, the AUC would be further calculated to evaluate the performance. Obviously, the AUC value of 1 represented a perfect prediction, while the AUC value of 0.5 represented a random prediction. Thereafter, simulation results show that NBLPIHMDA can achieve reliable AUCs of 0.8777 and 0.8958 ± 0.0027 under the frameworks of LOOCV and 5-fold CV, respectively, which indicated that NBLPIHMDA has satisfactory prediction performances.

**Figure 2:**
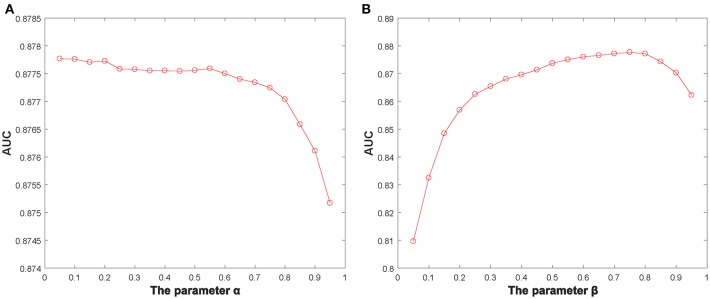
The AUCs achieved by NBLPIHMDA in LOOCV with different values of the parameters α and β, **(A)** β = 0.75, **(B)** α = 0.2.

Additionally, we identified several important parameters in NBLPIHMDA, such as the propagation probability α and the weighting factor β and so on. Hence, it is necessary to evaluate the impacts of these important parameters to the prediction performance of NBLPIHMDA. And as for evaluating the effects of the parameter α, we calculated the AUCs in framework of LOOCV with α varying from 0.05 to 0.95. The simulation results were shown in the following Figure 2, and as a result, it is obvious that NBLPIHMDA can achieve the highest AUC of 0.8777 while α = 0.2, which implies that while updating its label information, a node should retain more of its previous label information. Next, as for evaluating the effects of the parameter β, we calculated the AUCs in framework of LOOCV with β varying from 0.05 to 0.95. The simulation results were shown in the following Figure 2, and as a result, it is obvious that NBLPIHMDA can achieve the best prediction performance while β = 0.75, which means that the weight assigned to *W*_*LPD*_ should be greater than that of *W*_*LPM*_. Through analysis, the reason that why the weight assigned to *W*_*LPD*_ should be greater than that of *W*_*LPM*_ may be that the number of collected microbes is much larger than the number of diseases. Therefore, in NBLPIHMDA, we would set α = 0.2 and β = 0.75. And besides, according to our simulation results, the other two parameters *K* and *P* would be set to 5 and 10^−12^, respectively in this paper.

### 3.2. Comparison With Other Methods

**Figure 3:**
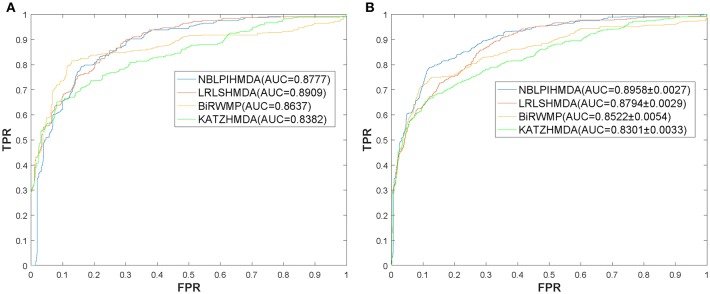
ROC curves and AUCs achieved by NBLPIHMDA, LRLSHMDA, BiRWMP, and KATZHMDA under LOOCV and 5-fold CV, respectively, **(A)** ROC curves and AUCs in LOOCV, **(B)** ROC curves and AUCs in 5-fold CV.

In this section, in order to attest the superior performance of our computational model NBLPIHMDA, we compared NBLPIHMDA with some state-of-the-art prediction methods such as KATZHMDA (Chen et al., 2016a), LRLSHMDA (Wang et al., 2017), and BiRWMP (Shen et al., 2018) under the frameworks of LOOCV and 5-fold CV, respectively. Here, KATZHMDA adopted the KATZ method and Gaussian interaction profile kernel similarity to infer potential microbe-disease associations by introducing the variable step number into a heterogeneous network. LRLSHMDA combined the Laplace's regular least squares classification with topological information of known microbe-disease association network to infer novel microbe-diseases associations. BiRWMP was a random walk based computational model for potential microbe-disease association prediction, and while comparing NBLPIHMDA with BiRWMP, in order to maintain consistency and accurate contrast, we replaced the Spearman correlations and symptom-based similarity of diseases adopted in BiRWMP with the Gaussian interaction profile kernel similarity. And as illustrated in the following Figure 3 and Table 1, simulation results show that NBLPIHMDA can achieve reliable AUCs of 0.8777 and 0.8958 ± 0.0027 in LOOCV and 5-fold CV, respectively, which are not only superior to the AUCs of 0.8382 and 0.8637 achieved by KATZHMDA and BiRWMP in LOOCV, but also superior to the AUCs of 0.8301 ± 0.0033 and 0.8522 ± 0.0054 achieved by KATZHMDA and BiRWMP in 5-fold CV simultaneously. While comparing with LRLSHMDA, although the AUC of 0.8777 achieved by NBLPIHMDA in LOOCV is not as good as the AUC of 0.8909 achieved by LRLSHMDA in LOOCV, however, the AUC of 0.8958 ± 0.0027 achieved by NBLPIHMDA in 5-fold CV is much better than the AUC of 0.8794 ± 0.0029 achieved by LRLSHMDA in 5-fold CV. Hence, it is obvious that the prediction performance of NBLPIHMDA outperforms that of these state-of-the-art prediction models mentioned above.

**Table 1:** The AUCs achieved by NBLPIHMDA, LRLSHMDA, BiRWMP, and KATZHMDA under the framework of LOOCV and 5-fold CV.

**Method**	**LOOCV**	**5-fold CV**
NBLPIHMDA	0.8777	0.8958 ± 0.0027
LRLSHMDA	0.8909	0.8794 ± 0.0029
BiRWMP	0.8637	0.8522 ± 0.0054
KATZHMDA	0.8382	0.8301 ± 0.0033

### 3.3. Case Study

We selected three diseases of widespread concern such as colorectal carcinoma, asthma, and COPD for case studies to investigate their pathogenic mechanism from the perspective of microbes. Interestingly, there are 10, 10, and 8 out of the top 10 predicted microbes could be validated, respectively for the three diseases by literature mining. The rankings of all potential microbe-disease pairs and the top ten related microbes of all diseases predicted by NBLPIHMDA are listed in Supplementary Tables 1, 5, respectively.

Asthma, a common chronic inflammatory pulmonary tracheal disease, affects more than 300 million people worldwide. Asthma usually occurs in childhood and is accompanied by a recurrent cough, wheezing, chest tightness, and dyspnea. Currently, there is no cure for asthma. Asthma is generally thought to be caused by a combination of genetic and environmental factors. In recent years, more and more studies have shown that microbes play an important role in the pathogenesis of asthma. Consequently, we conducted a case study of Asthma on our calculation model. And as illustrated in the following Table 2 and Supplementary Table 2, all of these top 10 predicted microbes interrelated with Asthma were verified to be correlative. For example, the colonization of *Clostridium difficile* (Ranking fist in the prediction list) at 1 month of neonatal age was closely related to asthma and eczema that occurred at 6–7 years of age (van Nimwegen et al., 2011). Compared with non-asthmatic healthy people, the increase of Firmicutes (Ranking second in the prediction list) mainly composed of streptococcal OTUs in patients with severe asthma is abnormally significant, and Actinobacteria (Ranking fifth in the prediction list) was found to have a lower proportion in asthmatic patients (Zhang et al., 2016). *Clostridium coccoides* (Ranking third in the prediction list) was confirmed to be significantly associated with a positive Asthma Predictive Index (API), and early fecal colonization with a *Clostridium coccoides* subcluster XIVa species could be an early indicator of asthma later in life (Vael et al., 2011). An increase in sensitivity to *Staphylococcus aureus* (Ranking fourth in the prediction list) enterotoxins in smokers with asthma could be considered as a marker of eosinophilic inflammation and exacerbation of asthma (Nagasaki et al., 2017). Long-term asthma patients have lower levels of Bifidobacteria (Ranking eighth in the prediction list) compared with patients who have recently been diagnosed with asthma (Hevia et al., 2016). The colonization of Bacteroides (Ranking tenth in the prediction list) at three weeks of infants was proved to be positively correlated with the positive asthma predictive index at 3 years of age, which may serve as the early indicator of asthma (Vael et al., 2008).

**Table 2:** The top 10 predicted microbes associated with asthma.

**Rank**	**Microbe**	**Evidence**
1	*Clostridium difficile*	PMID: 21872915
2	Firmicutes	PMID: 23265859
3	*Clostridium coccoides*	PMID: 21477358
4	*Staphylococcus aureus*	PMID: 17950502
5	Actinobacteria	PMID: 23265859
6	Clostridia	PMID: 21477358
7	Lachnospiraceae	Ciaccio et al. (2014)
8	Bifidobacterium	PMID: 26840903, PMID: 24735374
9	Lactobacillus	Gutkowski et al. (2011), PMID: 20592920
10	Bacteroides	PMID: 18822123, PMID: 29161087

Colorectal carcinoma, also known as colon cancer, is the third most common cancer, whose symptoms may include blood in the stool, abdominal pain, diarrhea, weight loss, prolonged fatigue, and flagging spirit. The prevalence of colorectal cancer is increasing year by year, about 4 to 5%. The risk is mainly caused by age, lifestyle, and genetic history. The gut microbiota also plays a related role, and disorder of the intestinal flora may affect the chronic inflammation mechanism and induce colon cancer. Hence we conducted a case study of Colorectal Carcinoma on our calculation model. And as illustrated in the following Table 3 and Supplementary Table 3, all of these top 10 predicted microbes interrelated with Colorectal Carcinoma were verified to be correlative. For example, infection with *Helicobacter pylori* (Ranking third in the prediction list), particularly CagA-positive flora, increased the risk of Colorectal cancer and gastric adenocarcinoma (Shmuely, 2001). By 16S rRNA gene denaturing gradient gel electrophoresis and ribosomal gene interval analysis (RISA), it was found that the abundance of *Clostridium difficile* (Ranking second in the prediction list) and *C. coccoides* (Ranking fourth in the prediction list) in colorectal cancer patients was significantly increased compared with healthy controls, which means that *C. cerevisiae* might have a potential effect on the induction of colorectal cancer (Leu et al., 2009). Protein a-containing *Staphylococcus aureus* (Ranking fifth in the prediction list) was used as an immunosorbent to perform *in vitro* immunoadsorption therapy on the plasma of patients with metastatic colorectal carcinoma and has produced good effects (Ishikawa et al., 2005). *Bifidobacterium lactis* (Ranking eighth in the prediction list) could prevent colorectal carcinoma by up-regulating the apoptosis reaction of colon carcinogens (Ray et al., 1982). The ingestion of *Lactobacillus casei* (Ranking tenth in the prediction list) had been shown in a large randomized clinical trial to reduce the incidence of moderate or severe heterogeneity tumors and to inhibit the atypia of colorectal tumors as a preventive measure (Scanlan et al., 2008).

**Table 3:** The top 10 predicted microbes associated with colorectal carcinoma.

**Rank**	**Microbe**	**Evidence**
1	Proteobacteria	PMID: 24603888
2	*Clostridium difficile*	PMID: 19807912
3	*Helicobacter pylori*	PMID: 11774957
4	*Clostridium coccoides*	PMID: 19807912
5	*Staphylococcus aureus*	PMID: 7074582
6	Actinobacteria	PMID: 24316595
7	Lachnospiraceae	PMID: 29985435
8	Bifidobacterium	PMID: 9111222
9	Haemophilus	PMID: 22761885
10	Lactobacillus	PMID: 15828052

COPD is a progressive obstructive pulmonary disease, which means it will get worse over time. It usually occurs in people over the age of 40, and the risk is the same for men and women. Smoking is the most common habit in patients with COPD. Besides, factors such as air pollution and genes will also increase the risk of COPD. Although treatment can be used to slow down the progression of the disease, there is no cure yet, and it is even more necessary to step up research on the pathogenesis of COPD. Therefore, in this section, COPD is selected as a case for study. And as shown in following Table 4 and Supplementary Table 4, 8 out of the top 10 predicted microbes associated with COPD were confirmed to be relevant. For instance, through the study, it was found that the serum *Helicobacter pylori*-specific IgG in COPD patients was significantly higher than that in the healthy control group, which implies that *Helicobacter pylori* (Ranking second in the prediction list) infection is closely related to COPD (Mammen and Sethi, 2016). In addition, there is evidence that IgE antibodies to *Staphylococcus aureus* (Ranking fifth in the prediction list) enterotoxin in patients with COPD are significantly higher than that in healthy subjects, suggesting that an immune response to this superantigen, *S. aureus* enterotoxin, may be a potential cause of chronic inflammation in COPD (Gencer et al., 2005). The increase of Actinomyces (Ranking sixth in the prediction list) and Proteobacteria was found to aggravate the deterioration of the pathogenesis of COPD (Rohde et al., 2004). *Bifidobacterium breve* (Ranking ninth in the prediction list) can serve as an anti-inflammatory agent to inhibit the expression and release of inflammatory mediators in COPD by inhibiting the activity of NF-kB induced by cigarette smoke which is the main cause of COPD (Mortaz et al., 2015).

**Table 4:** The top 10 predicted microbes associated with COPD.

**Rank**	**Microbe**	**Evidence**
1	*Clostridium difficile*	PMID: 15655746
2	*Helicobacter pylori*	PMID: 15733502
3	Firmicutes	PMID: 24591822
4	*Clostridium coccoides*	unconfirmed
5	*Staphylococcus aureus*	PMID: 7074582
6	Actinobacteria	PMID: 26852737
7	Clostridia	PMID: 26852737
8	Lachnospiraceae	unconfirmed
9	Bifidobacterium	PMID: 26317628
10	Bacteroides	PMID: 29709671

## 4. Discussion

There are numerous microbial communities inhabited in the human body, which is critical to human health. The relationship between human microbiome and diseases received much attention from both medical and bioinformatics community recently. However, traditional methods to detect their association is costly and labor-intensive. Thus, we proposed here a new computational model called NBLPIHMDA to infer potential microbe-disease associations. NBLPIHMDA first combined known microbe-disease associations in HMDAD and the Gaussian interaction profile kernel similarity to construct disease similarity network and microbe similarity network. It then conducted tag transmission on these two networks to obtain the predicted score of each microbe-disease pair. Under the framework of LOOCV and 5-fold CV, the AUCs reached 0.8777 and 0.8958 ± 0.0027, respectively, In addition, the case studies of asthma, colorectal carcinoma and COPD further demonstrated that NBLPIHMDA could provide valuable insights into the pathogenesis research.

It worth's noting that NBLPIHMDA has certain limitations. First of all, the HMDAD only curated hundreds of known associations between 39 diseases and 292 microbes, which is relatively small. The problem will be partially solved in the future when more associations between diseases and microbes are discovered. In addition, the Gaussian interaction profile kernel similarity of diseases and microbes is calculated based on the known microbe-disease associations, which will bias toward diseases with more known associations and microbes with more known associations. We believe that the bias can be reduced by integrating other effective similarity methods, such as disease semantic similarity, symptom-based disease similarity, and microbe functional similarity. The advancement of association prediction research in various fields of computational biology would also provide valuable insights into the development of microbe-disease association prediction, such as miRNA-disease association prediction (Chen and Huang, 2017; Chen et al., 2018a,b,c), lncRNA-disease association prediction (Chen and Yan, 2013), drug-target interaction prediction (Chen et al., 2015), and synergistic drug combinations (Chen et al., 2016b). Therefore, we will introduce some reliable technologies and optimization strategies in the future work to further improve the quality of the heterogeneous network and the prediction performance of NBLPIHMDA.

## Data Availability

The raw data supporting the conclusions of this manuscript will be made available by the authors, without undue reservation, to any qualified researcher.

## Author Contributions

LW and YW contributed to the conceptualization of the study. YW contributed to the methodology and writing, the reviewing, and editing of the manuscript. YW and HL contributed to the validation and data curation. LW, YW, HL, and XF contributed to the formal analysis. XF, DY, and JY contributed to the investigation. LW contributed to the resources, the project administration, and the funding acquisition. LW and JY contributed to the supervision of the study. All authors read and approved the final manuscript.

### Conflict of Interest Statement

The authors declare that the research was conducted in the absence of any commercial or financial relationships that could be construed as a potential conflict of interest.

## Supplementary Material

Supplementary Table 1 Final prediction result.

Supplementary Table 2 The top 10 predicted microbes associated with asthma.

Supplementary Table 3 The top 10 predicted microbes associated with colorectal carcinoma.

Supplementary Table 4 The top 10 predicted microbes associated with COPD.

Supplementary Table 5 The top ten related microbes of all diseases.
